# miR-146a deficiency in hematopoietic cells is not involved in the development of atherosclerosis

**DOI:** 10.1371/journal.pone.0198932

**Published:** 2018-06-14

**Authors:** Alberto del Monte, Ana B. Arroyo, María J. Andrés-Manzano, Nuria García-Barberá, María S. Caleprico, Vicente Vicente, Vanessa Roldán, Rocío González-Conejero, Constantino Martínez, Vicente Andrés

**Affiliations:** 1 Centro Nacional de Investigaciones Cardiovasculares Carlos III (CNIC), Madrid, Spain; 2 CIBER de Enfermedades Cardiovasculares (CIBER-CV), Murcia, Spain; 3 Servicio de Hematología y Oncología Médica, Hospital Universitario Morales Meseguer, Centro Regional de Hemodonación, Universidad de Murcia, IMIB-Arrixaca, Murcia, Spain; Goethe-Universitat Frankfurt am Main, GERMANY

## Abstract

**Background:**

Atherosclerosis involves activation of the IRAK1/TRAF6/NF-κB inflammatory cascade, which is negatively regulated by miR146a. Previous studies showed that the TT genotype of rs2431697, located near the *miR-146a* gene, drives lower miR-146a transcription and predicts adverse cardiovascular events in anticoagulated atrial fibrillation patients. Moreover, systemic miR-146a administration protects mice from atherosclerosis. Here we evaluated the ability of miR-146a expression in the hematopoietic component to regulate atherosclerosis in low-density lipoprotein receptor-null mice (*Ldlr*^*-/-*^).

**Methods and results:**

Lethally-irradiated *Ldlr*^*-/-*^ mice transplanted with bone marrow from wild-type or *miR-146a*-null mice were fed an atherogenic diet for 8 and 20 weeks. *Irak1*, *Traf6* and *MIR146A* expression were quantified in thoracic aorta by qRT-PCR and Western blot. Aortic plaque size and composition were characterized by Oil-Red staining and immunohistochemistry and leukocyte recruitment by intravital microscopy. Blood cell counts were similar in fat-fed *Ldlr*^*-/-*^mice with or without hematopoietic miR-146a expression. However, plasma cholesterol decreased in fat-fed *Ldlr*^*-/-*^mice transplanted with bone marrow deficient for miR-146a. Finally, aortic atherosclerosis burden and recruitment of leukocytes into the vessel wall were undistinguishable between the two groups, despite higher levels of *Irak1* and *Traf6* mRNA and protein in the aorta of fat-fed mice lacking hematopoietic miR-146a expression.

**Conclusions:**

miR-146a deficiency exclusively in hematopoietic cells modulates cholesterol levels in plasma and the expression of its targets in the artery wall of fat-fed *Ldlr*^*-/-*^ mice, but does not accelerate atherosclerosis. Atheroprotection upon systemic miR-146a administration may therefore be caused by specific effects on vascular cells.

## Introduction

Atherosclerosis is a complex inflammatory process involving several factors and cell types that interact in response to different forms of injury [1]. Together with endothelium and smooth muscle cells, hematopoietic cells play a central role in atherogenesis [2]. Indeed, a key early event in the inflammatory response during atherosclerosis is the adhesion of neutrophils to the vascular endothelium and their recruitment into the injured artery wall. The secretion of neutrophil-derived myeloperoxidase seems to be an important event in vascular injury [3]. More recent research has implicated the production of neutrophil extracellular traps (NETs) in atherogenesis [4], although further research is required to fully define the underlying mechanisms [5]. Other important hemostatic factors in atherosclerosis are monocytes/macrophages, important inflammatory and invasive cells that regulate plaque formation and necrosis [2,6]. As they infiltrate the artery wall, monocytes polarize into different macrophage subsets that play distinct roles in atherosclerosis [7].

MicroRNAs (miRNAs) are small, non-coding RNAs that posttranscriptionally regulate gene expression by promoting mRNA degradation and/or inhibiting mRNA translation [8,9]. Since their discovery, miRNAs have been implicated as key modulators of numerous physiological and pathological processes. Their role in cardiovascular disease has been extensively studied in recent years [10,11]. In particular, miR-146a has an anti-inflammatory function [12] and its expression is regulated by the single-nucleotide polymorphism (SNP) rs2431697; homozygote TT individuals have lower levels of miR-146a than GG individuals [13]. We recently demonstrated that the levels of miR-146a in monocytes may play an important role in the development of cardiovascular adverse events in patients with atrial fibrillation (AF), with rs2431697-TT patients displaying higher risk of developing adverse cardiovascular events [14]. In turn, monocytes from rs2431697-TT individuals have an increased pro-inflammatory response when subjected to inflammatory stress.

A role for miR-146a in atherosclerosis is suggested by its ability to negatively regulate several pro-inflammatory factors that promote disease progression, including Toll-like receptor 4 (TLR4), IL-1 receptor-associated kinase 1 (IRAK1), and TNF receptor-associated protein factor 6 (TRAF6) [15,16]. Moreover, low miR-146a levels in neutrophils are associated with carotid intima-media thickening in patients with systemic lupus erythematosus [17]. Recent mouse studies reveal that apolipoprotein E (ApoE) enhances miR-146a expression in monocytes and macrophages, suppressing NF-κB-mediated inflammation and atherosclerosis, and that systemic delivery of miR-146a mimetic attenuates monocyte/macrophage activation and atherosclerosis in the absence of plasma lipid reduction [18]. Given these recent studies and the relevance of leukocytes in all stages of atherosclerosis, we investigated whether miR-146a deficiency restricted to the hematopoietic compartment can aggravate the development of high-fat diet (HFD)-induced atherosclerosis in mice deficient for the low-density lipoprotein receptor (*Ldlr*^*-/-*^) at early (8 weeks) and late stages (20 weeks) of disease progression.

## Materials and methods

### Mice and bone marrow transplantation

Animal welfare, and all experimental and other scientific procedures with animals conformed to EU Directive 2010/63EU and Recommendation 2007/526/EC, enforced in Spanish law under Real Decreto 53/2013. In addition, the animal research included in this work was granted a formal ethics approval by the Committees in charge of animal welfare at Centro Nacional de Investigaciones Cardiovasculares Carlos III (OEBA-CNIC) and Universidad de Murcia (CEEA-UM), the Ethics Committee for Research from Universidad Autónoma de Madrid (OH-UAM), the committee form the Animal Protection Area of the Comunidad Autónoma de Madrid (CAM), and the Animal Health Service of the Department of Agriculture and Water from Región de Murcia (DAW-RM). The ethics approval numbers from the legally competent authorities are PROEX 134/14 (CAM) and A13150602 (DAW-RM). The animals were housed in cages in ventilated racks. Cages contained a bed of wood chips and nesting material, and objects for environmental enrichment. All the animals had ad libitum access to food and water. The maximum number of animals per cage was limited to 5. Mice were maintained under controlled environmental conditions (relative humidity: 45–65%; temperature: 20–24 °C) with light/darkness cycles of 12h/12h.

*miR-146a*^-/-^ mice (The Jackson laboratory, Bar Harbor, ME) were backcrossed for more than 8 generations in a C57BL/6J CD45.2 background. Irradiation and bone marrow (BM) transplantation was as described [19]. Briefly, *Ldlr*^*-/-*^ CD45.1 mice (8 to 10-week-old, Charles River) were irradiated with 2 doses of 6.5 Gy (10 minutes each, temperature: 37 °C) using a JL Shephed & Associates 1-68A irradiator with a source of 1000 curies of Cs-137. Next day, the animals were injected in the tail vein with 100μl of BM cells (7 x 10^6^, in saline) obtained from a pool of 4 femurs and 4 tibias of *miR-146a*^-/-^ or wild-type (wt) mice (both C57BL/6J CD45.2; 8-week-old). After 4 weeks on a standard diet, transplanted mice were fed HFD for 8 or 20 weeks (10.7% total fat, 0.75% cholesterol, S9167-E010, SSNIFF, Germany). Transplant efficiency was assessed as the percentage of donor CD45.2-immunoreactive cells in the blood of BM recipient mice, assessed by CD45.2 and CD45.1 immunostaining and flow cytometry.

Animal were all euthanized at the end of every experiment by inhalation of carbon dioxide gas.

### Quantification of atherosclerosis burden and immunohistopathology

All quantitative analyses were performed by an investigator blinded to genotype. Atherosclerosis burden was quantified as described [20]. Briefly, mice were euthanized in a CO_2_ chamber and the heart and aorta were extracted after *in situ* perfusion with PBS. Tissues were fixed with 4% paraformaldehyde/PBS overnight at 4°C. Atherosclerosis burden was quantified by computer-assisted morphometric analysis (SigmaScan pro 5, Systat Software Inc., San Jose, CA) of the aortic arch stained with Oil Red O (O0625, Sigma, 0.2% Oil Red O in 80% MeOH) and of hematoxylin/eosin-stained cross-sections from the aortic root; for each mouse, results were the mean of 3 cross-sections. The area of the necrotic core in atheroma plaques was quantified by analyzing hematoxylin/eosin-stained aortic cross-sections (mean of 3 cross-sections per mouse).

### Blood cell counting and biochemical parameters

Blood was extracted from the facial vein and was analyzed to quantify and identify circulating blood cell populations using the PENTRA 80 hematology platform (HORIBA Medical, Madrid, Spain). Plasma was isolated by centrifugation of whole blood (2000*g*, 15´ at room temperature), and lipid profile was obtained using DIMENSION RxL MAX (Siemens, Munich, Germany).

### Intravital microscopy

Male *Ldlr*^*-/-*^ mice transplanted with BM wt or BM *miR-146a*^-/-^ fed HFD for 20 weeks after transplantation were used for cremaster muscle intravital microscopy as previously described [21]. Briefly, once exteriorised, the cremaster muscle was placed onto an optical clear viewing pedestal, cut longitudinally with a high temperature surgical cautery (Lifeline Medical) and held extended at the corners of the exposed tissue using surgical suture. Ten min before image acquisition, antibodies (0.5 μg) antiCD4 (Tonbo), anti-Ly6G (Biolegend) and anti-Ly6C (eBioscience) were injected to label myeloid cell subsets. To maintain the correct temperature and physiological conditions, exposed tissues were perfused continuously with 37°C pre-warmed Tyrode’s buffer solution (139 mM NaCl, 3 mM KCl, 17 mM NaHCO_3_, 12 mM Glucose, 3 mM CaCl_2_ and 1 mM MgCl_2_). The cremasteric microcirculation was visualized using an AXIO Examiner Z.1 work station (Zeiss) mounted on a 3-dimensional motorized stage (Sutter Instrument) and equipped with a CoolSnap HQ2 camera (Photometrics). An APO 20x NA 1.0 water-immersion objective was used. Slidebook software 5.0 (Intelligent Imaging Innovations) was employed for acquisition and image processing. Three randomly-selected arterioles and venules were analysed per mouse, and T cells/monocytes/neutrophils were considered adherent when no rolling was observed using a reference line perpendicular to blood flow for at least 30 seconds.

### Quantitative real-time-PCR (qRT-PCR)

Total RNA was isolated from thoracic aortas using RNAzol reagent (Molecular Research Center Inc) and was retrotranscribed (100 ng) using the SuperScript^™^ III First-Strand Synthesis System (ThermoFisher). MiR-146a cDNA was synthesized using individual miRNA-specific RT primers and the TaqMan^®^ MicroRNA Reverse Transcription Kit (ThermoFisher). cDNA was amplified using TaqMan^®^ probes (*Irak1*: Mm01193538_m1, *Traf6*: Mm00493836_m1) together with TaqMan^®^ Universal PCR Master Mix, No AmpErase^®^ UNG (ThermoFisher). The 2^-ΔCt^ method was used to calculate the relative abundance of mRNA compared with endogenous actin (*Actb*: Mm02619580_g1) mRNA and of miRNA compared with U6 for miR-146a (Ct = Threshold Cycle; ΔCt = Ct sample gene-Ct endogenous control).

### Western blotting

Protein lysates were obtained from thoracic aortas of BM *miR-146a*^-/-^and BM wt transplanted mice fed with a HFD for 20 weeks. Tissue homogenization was performed using TissueLyser (Qiagen). Normalization was based on the amount of protein loaded. Total protein (20 μg) was separated by SDS-PAGE (8%) in reducing conditions. Gels were transferred onto PVDF membranes (Amersham Hybond P 0.45, GE Healthcare, Barcelona Spain) and blocked for 1 hour in 5% w/v BSA-TBS. Membranes were incubated overnight at 4°C with primary antibodies against human Traf6 (#sc-8409, 1:1,000, Santa Cruz, Heidelberg, Germany), Irak1 (D51G7, 1:1,000, Cell Signalling Technology, Leiden, The Netherlands), and β-actin (AC-15 clone, 1:5,000, Sigma-Aldrich, Madrid, Spain). After washing, membranes were incubated with secondary antibodies labeled with peroxidase (1:10,000, GE Healthcare, Barcelona, Spain). ECL Prime Detection Kit and ImageQuant LAS 4000 Imager (GE Healthcare, Barcelona, Spain) were used for protein detection. Densitometric analyses were performed with ImageJ software (http://rsb.info.nih.gov/ij/).

### Statistical analysis

Data are presented as mean ±SEM. In experiments with 2 groups, statistical significance was evaluated using a 2-tailed, unpaired Student’s *t*-test. For experiments comparing more than one group, a 1 or 2-way ANOVA with the Bonferroni post hoc tests were performed (GraphPad Prism software, La Jolla, CA). Differences were considered statistically significant at P<0.05.

## Results

### Genetic disruption of miR-146a in hematopoietic cells does not increase diet-induced atherosclerosis in Ldlr^-/-^ mice

**Fig 1:**
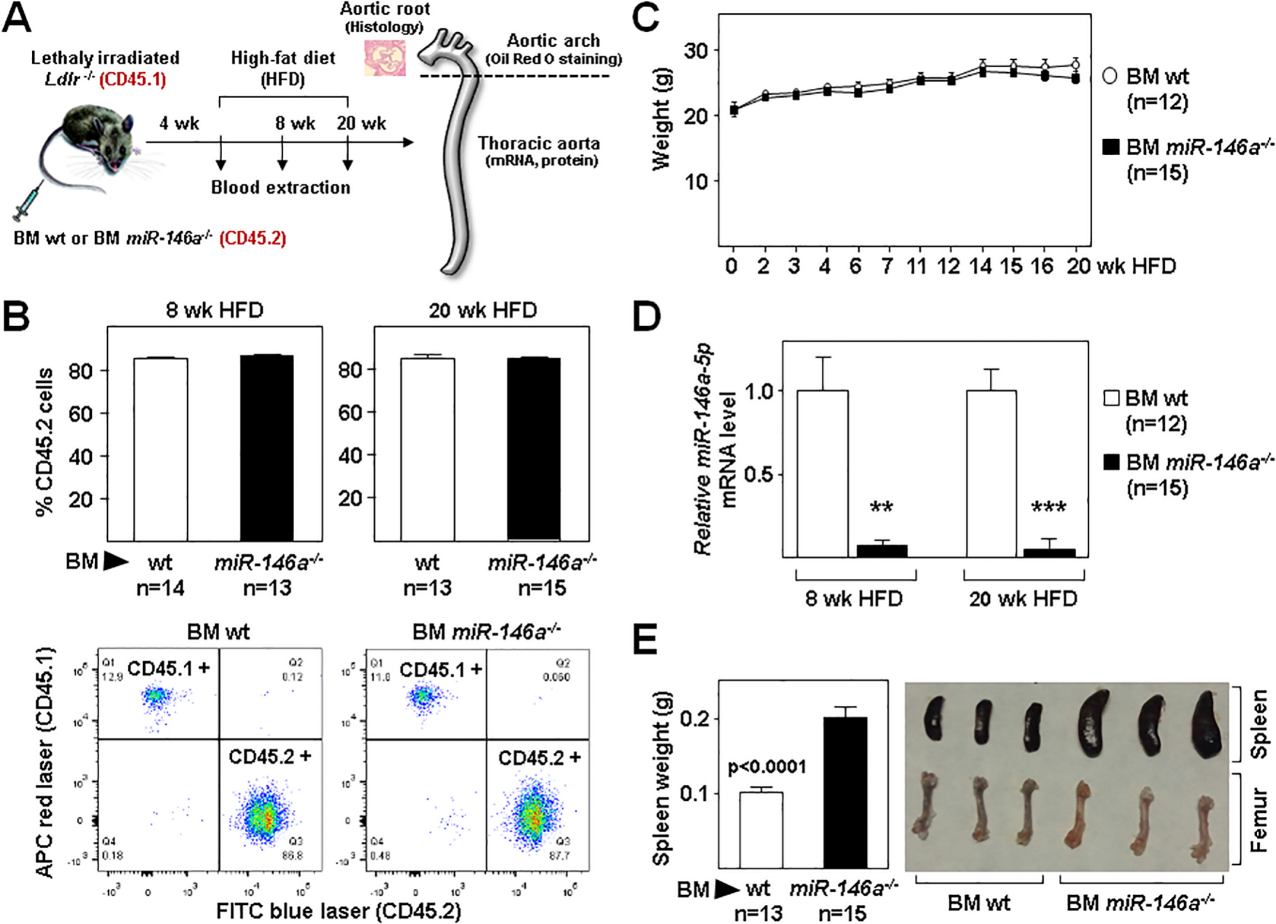
Study design and efficiency of bone marrow transplantation. **(A)** Lethally-irradiated *Ldlr*^*-/-*^ mice (CD45.1, 8 to 10-week-old) were transplanted with cells obtained from bone marrow (BM) of wt or *miR-146a*^-/-^ mice (both CD45.2). After 4 weeks of recovery, mice were challenged with a high-fat diet for 8 and 20 weeks. Blood and aorta were collected for biochemical and expression studies and to quantify atherosclerosis burden. **(B)** Quantification of transplant efficiency in both groups of mice as determined by flow cytometry of blood extracted 1 month after BM transplant. Donor and host cells are CD45.2- and CD45.1-immunoreactive, respectively. Representative flow cytometry profiles are shown below the chart. **(C)** Body weight at different times after the onset of HFD. **(D)** Pre and post diet miR-146a levels in blood leukocytes were measured by qRT-PCR. The 2^-ΔCt^ method was used to calculate miRNA abundance relative to U6 (Ct = Threshold Cycle; ΔCt = Ct sample gene − Ct endogenous control). **: p<0.01, ***: p<0.001 BM wt vs BM *miR-146a*^-/-^ at both time points. (**E**) Spleen from BM wt or BM *miR-146a*^-/-^ mice were weighed after 20 weeks of HFD. Shown are representative images of spleens and femurs.

To assess whether miR-146a deficiency restricted to the hematopoietic compartment affects atherosclerosis, *Ldlr*^*-/-*^ mice (CD45.1 background) were transplanted with BM from *miR-146a*^*-/-*^ (BM *miR-146a*^-/-^) or wt (BM wt) mice (both CD45.2 background) (Fig 1A). After one month, *Ldlr*^*-/-*^ mice were examined to evaluate transplant efficiency by quantifying CD45.1 and CD45.2 expression in circulating blood cells. These studies revealed similar transplant efficiency in both experimental groups (Fig 1B). Transplanted mice were then fed a HFD. Analysis of BM *miR-146a*^-/-^and BM wt mice fed normal chow (pre-diet) and HFD (post-diet) revealed no statistically significant between-group differences in body weight (Fig 1C). MiR-146a levels in blood leukocytes were significantly decreased in BM *miR-146a*^-/-^ transplanted mice before and after HFD (Fig 1D). Moreover, in agreement with previously published results [22], we found enlarged spleens and pale BM in *miR-146a*^-/-^ transplanted mice (Fig 1E).

**Fig 2:**
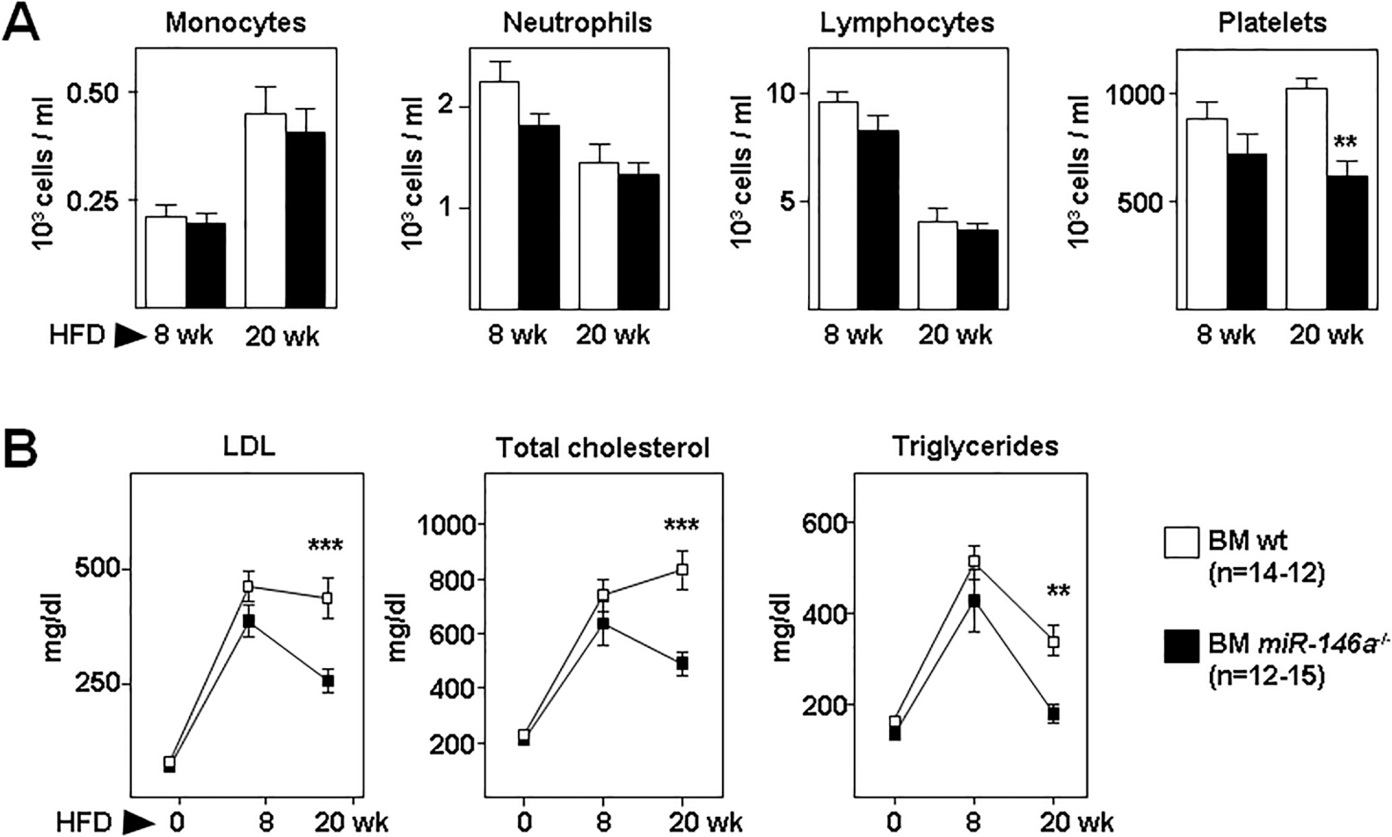
Hematology and plasma lipids in transplanted *Ldlr*^-/-^ mice. Lethally-irradiated *Ldlr*^*-/-*^ mice (CD45.1, 8 to 10-week-old) were transplanted with cells obtained from bone marrow (BM) of wt or *miR-146a*^-/-^ mice (both CD45.2). After 4 weeks of recovery, mice were challenged with a high-fat diet (HFD) for 8 and 20 weeks. **(A)** Blood cell populations from BM wt or BM *miR-146a*^-/-^ mice after HFD. **(B)** Plasma levels of LDL, total cholesterol, and triglycerides at 0, 8 and 20 weeks of HFD. **: p<0.01, ***: p<0.001 BM wt vs BM *miR-146a*^-/-^ at 20 weeks of HFD.

Circulating blood cell counts and populations were similar in both groups of mice, except for platelets that were significantly reduced in BM *miR-146a*^-/-^ after 20 weeks (Fig 2A). Plasma levels of low-density lipoprotein cholesterol (LDL), total cholesterol, and triglycerides increased significantly in both groups of mice fed HFD for 8 weeks compared with their pre-diet values, but no statistically-significant between-group differences were observed (Fig 2B). However, after 20 weeks of HFD, plasma lipid levels significantly decreased in BM *miR-146a*^-/-^ mice compared with BM wt mice (Fig 2B). Thus, genetic disruption of miR-146a in hematopoietic cells did not affect body weight, nor blood cell counts and populations, but reduced plasma lipids after 20 weeks of HFD.

**Fig 3:**
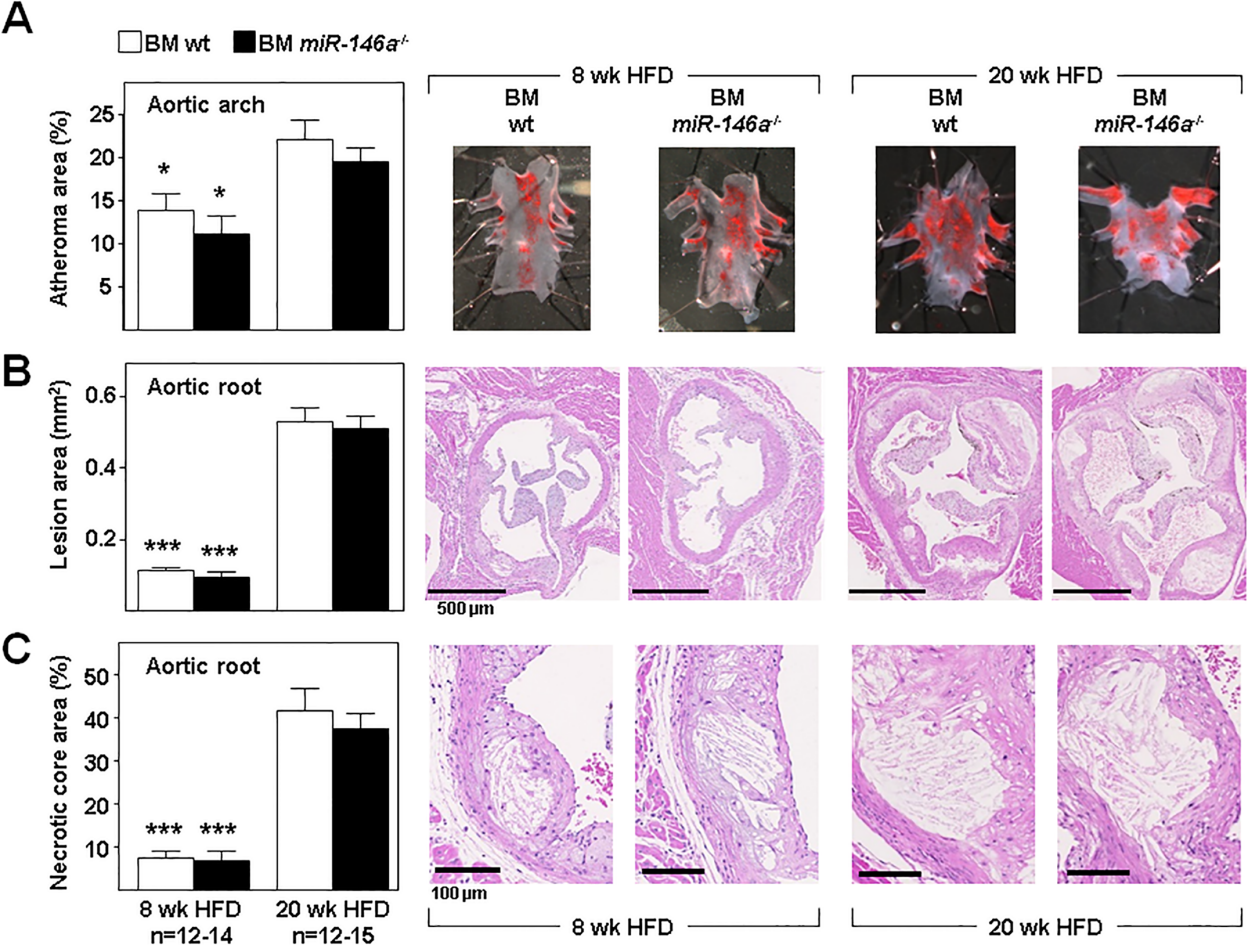
Ablation of miR-146a gene in hematopoietic cells does not affect diet-induced atherosclerosis in *Ldlr*^-/-^ mice. Lethally-irradiated *Ldlr*^*-/-*^ mice (CD45.1, 8 to 10-week-old) were transplanted with cells obtained from bone marrow (BM) of wt or *miR-146a*^-/-^ mice (both CD45.2). After 4 weeks of recovery, mice were challenged with a high-fat diet (HFD). Mice were euthanized after 8 or 20 weeks of HFD and tissues were harvested for immunohistopathological characterization of atherosclerotic lesions. Representative images are shown in each panel. **(A)** Percentage of aortic arch are occupied by atheroma visualized by *en face* Oil Red O staining. **(B)** Quantification of atheroma size in hematoxylin/eosin-stained sections of the aortic sinus. **(C)** Area of atherosclerotic lesions occupied by necrotic cores quantified in histological sections from the aortic sinus. *: p<0.05, ***: p<0.001 vs same BM genotype at 20 weeks of HFD.

We next quantified atherosclerosis burden in fat-fed mice by planimetric analysis of whole-mount Oil Red-O–stained aortic arch (Fig 3A) and hematoxylin/eosin-stained cross-sections through the aortic root (Fig 3B) after 8 and 20 weeks of HFD. As expected, these studies revealed increased atherosclerosis burden in mice of both groups at 20 weeks of HFD compared with 8 weeks of HFD; however, atherosclerosis development at both times of HFD was similar in BM *miR-146a*^-/-^ and BM wt mice in all regions (Fig 3). Similarly, lack of miR-146a in hematopoietic cells did not affect the formation of necrotic cores in the atherosclerotic plaques of mice fed HFD for 8 and 20 weeks (Fig 3C).

### Lack of miR-146a in hematopoietic cells does not augment adhesion of leukocytes in fat fed Ldlr-/- mice

**Fig 4:**
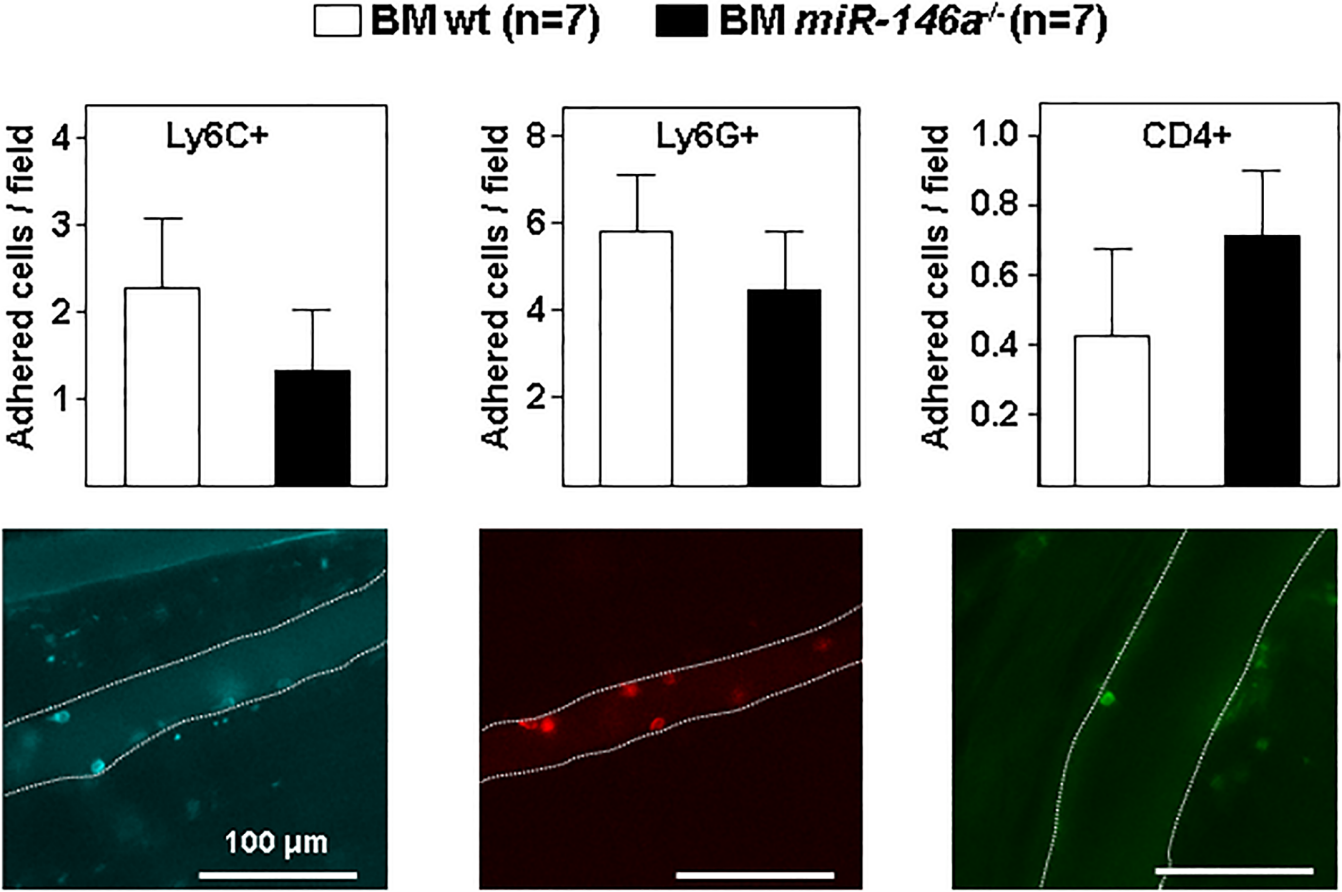
Deletion of miR-146a gene in hematopoietic cells does not increase adhesion in *Ldlr*^-/-^ mice fed HFD. Lethally-irradiated *Ldlr*^*-/-*^ mice (CD45.1, 8 to 10-week-old) were transplanted with cells obtained from bone marrow (BM) of wt or *miR-146a*^-/-^ mice (both CD45.2). After 4 weeks of recovery, mice were challenged with a high-fat diet (HFD) for 20 weeks and analyzed by intravital microscopy in the cremaster muscle. Images are representative for Ly6C^+^ monocytes, Ly6G^+^ neutrophils, and CD4^+^ T cells adhered to the wall of cremasteric venules. Dashed lines mark the boundaries of the venules.

We next evaluated if miR-146a deficiency in the BM compartment may affect the recruitment and adhesion of leukocytes to the inflamed vessel wall of *Ldlr*^*-/-*^ mice fed HFD for 20 weeks. For this purpose, we analyzed by intravital microscopy the *in vivo* adhesion of labeled leukocytes (Ly6C^+^ monocytes, Ly6G^+^ neutrophils, and CD4^+^ T cells) to the endothelium of cremaster muscle venules. These studies revealed no differences in the adhesion of the different subtypes of leukocytes between BM *miR-146a*^-/-^ and BM wt fat-fed *Ldlr*^*-/-*^ mice (Fig 4).

### Expression of miR-146a targets in fat-fed mice

**Fig 5:**
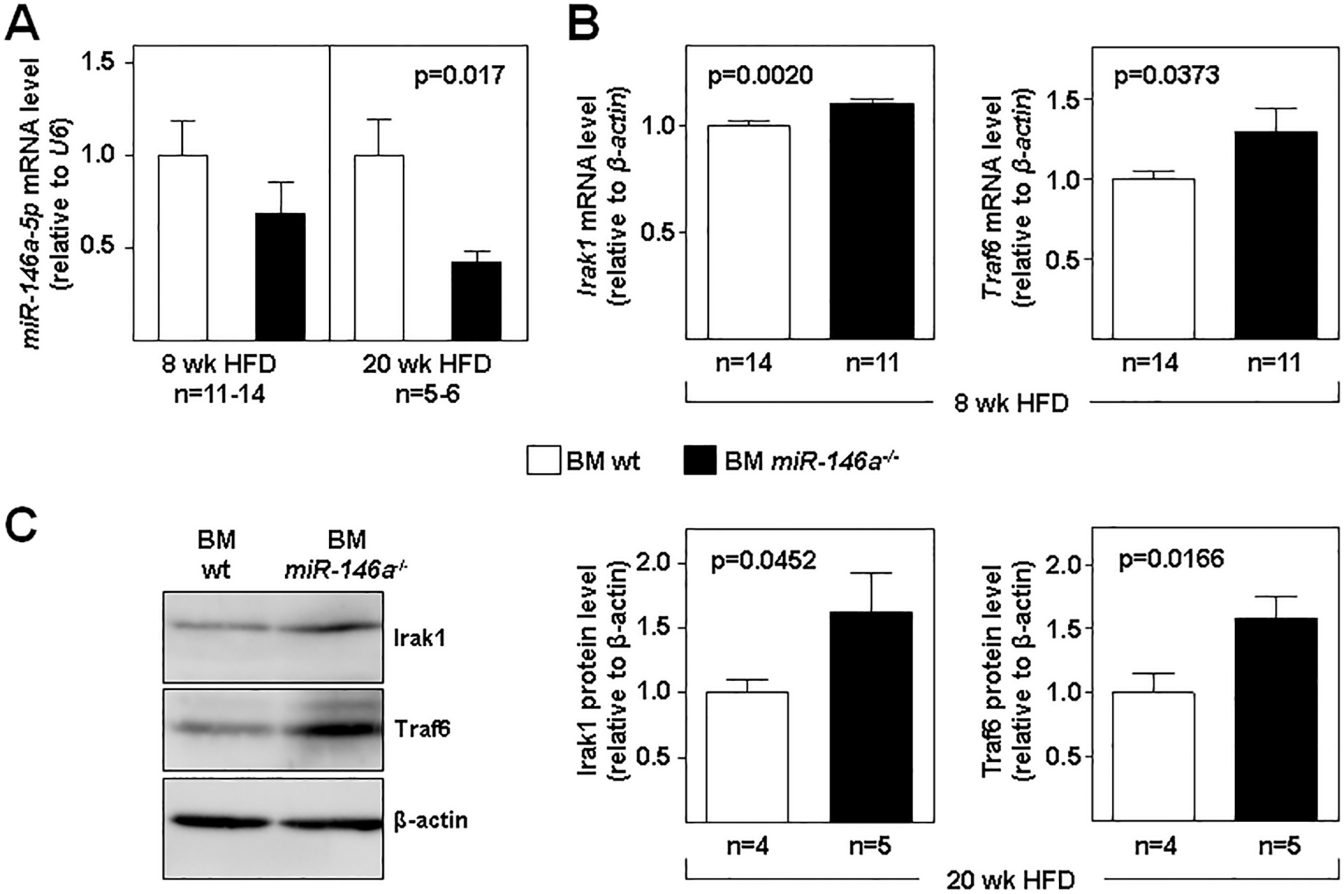
Expression of *miR-146a* targets in fat-fed mice. Lethally-irradiated *Ldlr*^*-/-*^ mice (CD45.1, 8 to 10-week-old) were transplanted with cells obtained from bone marrow (BM) of wt or *miR-146a*^-/-^ mice (both CD45.2). After 4 weeks of recovery, mice were challenged with a high-fat diet (HFD) for the indicated times. **(A)** miR-146a expression was measured by qRT-PCR in total RNA extracted from the thoracic aorta of mice fed high-fat diet (HFD) for 8 weeks (individual thoracic aortas) and 20 weeks (pools of 3 thoracic aortas per samples). (**B**) miR-146a targets *Irak1* and *Traf6* mRNA levels were measured by qRT-PCR after 8 weeks of HFD. The 2^-ΔCt^ method was used to calculate miRNA/mRNA abundance relative to endogenous control expression (U6 in A; β-actin in B) (Ct = Threshold Cycle; ΔCt = Ct sample gene − Ct endogenous control). **(C)** Representative western blot and quantification of Irak1 and Traf6 protein levels (normalized to β-actin) in thoracic aorta of mice receiving BM wt or BM *miR-146a*^-/-^ after 20 weeks of HFD (pools of 3 thoracic aortas per sample). *: p<0.05, **: p<0.01 BM wt vs BM *miR-146a*^-/-^ at both time points.

Thoracic aortas were homogenized and RNA extracted. As expected, the level of miR-146a was lower in tissue from fat-fed BM *miR-146a*^-/-^ mice compared with BM wt mice at both times of HFD (Fig 5A). Accordingly, mRNA levels of the miR-146a targets *Traf6* and *Irak1* were significantly higher in thoracic aortas of BM *miR-146a*^-/-^ mice after 8 weeks of HFD (Fig 5B). Similarly, Traf6 and Irak1 protein levels were significantly higher in thoracic aortas of BM *miR-146a*^-/-^ mice after 20 weeks of HFD (Fig 5C).

## Discussion

There is increasing evidence connecting miRNAs with the molecular pathophysiological processes leading to atherosclerosis (reviewed in [11,23]). Inflammation plays a pivotal role in all stages of atherogenesis, from its initiation until plaque rupture [1,6]. MiR-146a was one of the first miRNAs implicated in inflammatory processes [12]. Through the regulation of different targets of the TLR/NF-κB pathway, miR-146a acts as a negative feedback regulator of the innate immune response [24]. However, the implication of this miRNA in atherosclerosis has been addressed only recently, and several questions remain. Defining the cells in which miR-146a exerts its action will improve understanding of the role of this miRNA in atherogenesis. Recently, Li *et al*. reported that the anti-atherogenic action of ApoE, which is highly expressed in monocytes and macrophages, is mediated through increased expression of the transcription factor PU.1, resulting in enhanced miR-146a expression in monocytes/macrophages and suppression of NF-κB-driven inflammation [18]. These authors also found that systemic delivery of miR-146a mimetic inhibits monocyte/macrophage activation and atherosclerosis development [18]. We observed that lack of miR-146a in the hematopoietic compartment does not affect atherosclerotic plaque formation in *Ldlr*^-/-^ mice at early and late stages of disease progression (8 and 20 weeks of HFD). This finding was unexpected because inhibition of the main miR-146a TLR/NF-κB pathway targets in monocytes/macrophages seemed to be protective [18]. Moreover, inhibition of NF-κB activation in macrophages increases atherosclerosis in *Ldlr*^-/-^ mice, although surprisingly the opposite occurs in endothelial cells [25]. This was further demonstrated in a model of NEMO/IKKg deletion and upon endothelial cell-specific expression of a dominant-negative IkBa mutant [26]. Different roles of a TLR/NF-κκB pathway component in endothelium *vs*. myeloid cells have also been described for TRAF6 by Polykratis *et al* [27]. These authors showed that endothelial-cell-expressed TRAF6 induces the expression of proinflammatory mediators that enhance the adhesion and recruitment of macrophages to the endothelium, whereas in myeloid cells the opposite occurs, with TRAF6 deficiency favoring the development of atherosclerosis by inhibiting the expression of IL-10 [27]. Our results demonstrate that decreasing the levels of miR-146a in the hematopoietic compartment does not increase atherosclerosis at both early (8 weeks HFD) and late (20 weeks HFD) stages of disease development. Of note, Cheng *et al*. recently showed no differences in atherosclerosis burden in the aortic arch of *Ldlr*^*-/-*^ mice transplanted with BM *miR-146a*^*-/-*^and fed HFD for 4 weeks; however, they found a significant decrease in plaque burden in BM *miR-146a*^*-/-*^ *Ldlr*^*-/-*^ mice after 12 weeks of HFD [22]. Importantly, these authors observed unchanged plaque burden in the descending thoracic aorta and aortic root at both time points with HFD. Among other reasons, Cheng *et al*. suggested that low levels of cholesterol after 12 weeks of HFD might explain this atheroprotective role in aortic arch provoked by a miR-146a deficiency in the hematopoietic compartment [22]. We also observed a significant decline in cholesterol after 20 weeks of HFD in BM *miR-146a*^*-/-*^, but this was not sufficient to reduce plaque burden under our experimental conditions. Consistent with our results showing no differences in atherosclerosis development in fat-fed BM *miR-146a*^*-/-*^ *Ldlr*^*-/-*^ mice, our intravital microscopy studies revealed no differences in vascular leukocyte recruitment upon *miR-146a*^*-/-*^ deficiency in BM cells.

Paradoxically it has also been described that inhibiting miR-146a in endothelial cells may increase the expression of some of its targets, such as TRAF6, thus enhancing NF-κB activation and promoting atherogenesis [28]. Indeed, Cheng *et al*. also demonstrated that mice deficient for miR-146a except in the hematopoietic compartment have a significant increase in atherosclerotic plaque burden in comparison with mice lacking miR-146a in all cells [22]. In line with these results, endothelium-specific delivery of miR-146a-loaded E-selectin-targeting microparticles reduced atherosclerosis in *ApoE*^-/-^mice [29]. In their study of the effect of systemically delivered miR-146a mimetics, Li *et al*. examined expression of endogenous miR-146a in leukocytes, but not in the aorta [18]. It is therefore possible that this treatment increased miR-146a levels predominantly in endothelium, as reported for *in vivo* injection of miRNA mimetics in other studies [30,31]. Thus, the potential beneficial effect of miR-146a in atherosclerosis might be due in part to increased expression in endothelial cells. Thus, despite observing no differences in atherosclerosis burden, we found above-normal expression of specific targets of miR-146a such as Traf6 and Irak1 in the aorta of BM 146a^-/-^ mice, which exhibited low level of miR-146a expression. Since levels of miR-146a expression in non-hematopoietic cells are unaffected by BM transplantation, differences in the expression of *miR-146a* and its two targets Traf6 and Irak1 might be due to variations in infiltrated leukocytes. Since *miR-146a* deficiency does not increase leukocyte adhesion, leukocyte infiltration should be similar in BM *miR-146a*^*-/-*^ and BM *wt* mice. Thus, *miR-146a* deficiency in hematopoietic cells affects *miR146a* targets in the inflamed aorta but not sufficiently to aggravate atherosclerosis development in *Ldlr*^-/-^ mice. Future studies using conditional *miR146a*-deficient mice for endothelium or vascular smooth muscle cells may help to unequivocally establish this hypothesis. Additionally, since miR-146a-/- mice still express miR-146b, which shares most of the targets with miR-146a, we cannot discard that miR-146b has a compensatory effect in the setting of atherosclerosis model [32]. In conclusion, our results strongly suggest that miR-146a expressed in the hematopoietic compartment plays a negligible role in atherosclerosis development in *Ldlr*^-/-^ mice, although its ablation increases in the aorta the proinflammatory state promoted by an atherogenic diet. Identifying how expression of this miRNA limits atherosclerosis remains of high interest. It will therefore be important to develop in vivo models in which to investigate the cells, with special attention to endothelial cells, in which miR-146a exerts its anti-atherogenic function. Finally, investigating the effect of low levels of miR-146a in humans due for example to rs2431697 in atherosclerosis may be of interest to evaluate the clinical impact of a partial miR-146a deficiency.
